# Conjoint analysis of methylation, transcriptomic, and proteomic profiles in pemphigus vulgaris

**DOI:** 10.1186/s13023-024-03458-6

**Published:** 2024-11-26

**Authors:** Xiaojia Luo, Jianting Ouyang, Fuqiong Jiang, Yaozhong Zhang, Yuan Wang, Yongzhuo Wu, Lingyu Hu

**Affiliations:** 1 Department of Dermatology, The Second Affiliated Hospital of Kunming Medical University, No.374 Dianmian Road, Wuhua District, Kunming, Yunnan Province 650101 China; 2 Department of Dermatology, Haikou Branch hospital of the Second Affiliated Hospital of Kunming Medical University, Kunming, Yunnan 650014 China

**Keywords:** Expression profile, Methylation, Pathogenesis, Pemphigus, Platelet activation, Proteomics

## Abstract

**Background:**

The underlying pathogenesis of pemphigus vulgaris, an autoimmune skin disorder, remains incompletely understood. An integrative analysis comprising DNA methylation, mRNA expression, and proteomic data in patients with pemphigus vulgaris was conducted to identify potential pathogenic contributors and explore the molecular mechanisms involved in its pathogenesis.

**Results:**

The analysis revealed differentially methylated regions (DMRs) in the promoter, exon, intron, and downstream regions in the peripheral blood DNA of patients with pemphigus vulgaris. Associations between methylation levels and both transcriptomic and proteomic profiles revealed that differentially expressed genes between patients with pemphigus vulgaris and healthy controls were primarily linked to biological functions such as platelet activation and coagulation, cellular adhesion, and immunoglobulin binding. Kyoto Encyclopedia of Genes and Genomes (KEGG) pathway enrichment analysis highlighted notable pathway abnormalities, including those related to platelet activation, focal adhesions, tight junctions, and infectious inflammatory responses. Notably, genes such as *FGA* (fibrinogen alpha chain), *VWF* (von Willebrand factor), and *ACTG1* (actin gamma 1) were dysregulated, with a prominent role in platelet activation.

**Conclusion:**

The dysregulation of genes such as *FGA*, *VWF*, and *ACTG1* suggests that alterations in their transcription and expression may contribute to the pathogenesis of pemphigus vulgaris.

## Background

Pemphigus is an autoimmune blistering disease affecting the skin and mucous membranes, characterized by intraepidermal blisters resulting from the loss of keratinocyte cell-cell adhesion. This loss is linked to both in vivo bound and circulating IgG autoantibodies that target the cell surface of keratinocytes. Although the precise causes of pemphigus remain unclear, the disease has been associated with environmental factors, infections, certain medications, and tumors, as well as specific human leukocyte antigen class II risk alleles, which may increase susceptibility [1]. 

Desmogleins (Dsg) are protein components of desmosomes, major epidermal adhesion structures. Desmogleins have four isoforms (Dsg1-4). Expression of Dsg1 and Dsg3 is basically restricted to stratified squamous epithelia, where blisters are formed in pemphigus, while Dsg2 is expressed in all desmosome-possessing tissues, including simple epithelia and myocardium. Dsg4 plays an important adhesive role primarily in hair follicles. In pemphigus, the basic pathophysiology involves disruption of the adhesive function of Dsg by antibodies, leading to keratinocyte cell-cell adhesion loss and subsequent blister formation.

However, the exact sequence of events following antibody binding is not completely understood. One hypothesis is that antibodies interfere directly with the adhesive function of Dsg via steric hindrance, that is, the spatial binding of autoantibodies prevents desmoglein interactions between cells. Alternatively, antibody binding might disrupt cell-cell adhesion in keratinocyte cultures by activating signal transduction pathways [2]. Moreover, abnormalities in immune responses and epigenetic modifications are thought to play important roles in the production of antibodies [3, 4]. 

DNA methylation, particularly within CpG islands, causes transcriptional inactivation of tumor suppressor genes, a process closely associated with human development and the pathogenesis of neoplastic diseases, thus establishing DNA methylation as an integral aspect of epigenomics. This epigenetic modification plays a key role in the regulation of gene expression and can affect both gene transcription and subsequent protein expression. Alterations in protein expression can in turn, manifest as specific clinical symptoms. An increasing body of research indicates that aberrant DNA methylation—defined as a statistically significant increase or decrease in methylation levels compared to those observed in a healthy control group—may be closely related to the pathogenesis of various inflammatory skin diseases such as psoriasis [5], systemic lupus erythematosus (SLE) [6], atopic dermatitis, and systemic scleroderma, as well as other conditions such as androgenetic alopecia, chronic eczema, and pemphigus [7–9]. However, the specific biological functions of the aberrant methylation pattern in these diseases were not elaborated upon in these studies [6, 7]. 

Pemphigus vulgaris is the most common type of pemphigus. In this study, the molecular mechanisms underlying pemphigus vulgaris were examined at the DNA, RNA, and protein levels by using a combination of whole-genome bisulfite sequencing (WGBS), whole-gene expression profiling, and proteomic analysis. The findings aim to provide an empirical foundation for the future development of molecular diagnostic markers and therapeutic targets.

## Materials and methods

### Study participants and specimens

The case group in this study consisted of patients with pemphigus vulgaris who sought treatment at the Second Affiliated Hospital of Kunming Medical University, China, in 2020 (*n* = 4), including two males and two females. The patients were diagnosed with pemphigus vulgaris based on clinical presentations, skin pathology, and direct skin immunofluorescence examination, with all patients in the active disease stage and a moderate Pemphigus Disease Area Index (PDAI) score (8–24 points). The control group (*n* = 4) comprised healthy age- and gender-matched individuals with no history of autoimmune disease, tumors, or infections, comprising two males and two females.

All collected specimens were immediately frozen in liquid nitrogen, stored at -80 °C, and transported on dry ice.

## Materials and methods

Comprehensive and precise identification of DNA methylation patterns across the entire genome was achieved at a single-base resolution level through the integration of whole-genome bisulphite sequencing (WGBS) with bisulfite conversion and next-generation sequencing technology. Total whole blood DNA was extracted using the QIAamp Fast DNA Tissue Kit (Qiagen, Dusseldorf, Germany). Bisulfite sequencing libraries were prepared using the Acegen Bisulfite-Seq Library Prep Kit (Acegen, Cat. No. AG0311) as per the manufacturer’s instructions. Briefly, genomic DNA spiked with unmethylated Lambda DNA was fragmented by sonication to an average size of 200–500 bp, then subjected to end-repair, 5’-phosphorylation, 3’-dA-tailing, and ligation with 5-methylcytosine-modified adapters. After bisulfite treatment, the DNA was amplified by 10 cycles of PCR. The resulting libraries were analyzed using an Agilent 2100 Bioanalyzer and subsequently sequenced on HiSeq4000 platforms with a 2 × 150 bp paired-end sequencing protocol.

Trizol (Invitrogen) reagent was used to extract and stabilize total RNA from the serum samples of patients and controls. RNA quantification was performed with a NanoDrop ND-2000 (Thermo Scientific), and RNA integrity was assessed using an Agilent Bioanalyzer 2100 (Agilent Technologies). To ensure optimal probe labeling efficiency and reliable chip hybridization results, RNA purification was carried out with the QIAGEN RNeasy Kit [10]. From each sample, 250 ng of purified total RNA was labeled and amplified. First, the AffinityScript-RT kit and Oligo dT-Promoter Primer were used for reverse transcription of RNA to synthesize the first strand of cDNA, followed by the addition of an anti-sense promoter to generate the second strand of cDNA. T7RNA polymerase was added to amplify the second strand of cDNA, resulting in complementary RNA (cRNA). Subsequently, the cRNA was labeled with the fluorescent dye Cyanine-3-CTP (Cy3) and purified using the QIAGEN RNeasy Kit. Finally, it was hybridized by rolling at 65℃ for 17 h. Following elution, the original image was scanned with an Agilent Scanner G5761A (Agilent Technologies).

The isobaric tag for relative and absolute quantitation (iTRAQ) was used to quantitatively analyze the changes in protein expression in peripheral venous blood samples from patients with pemphigus vulgaris and the control group.

The experiments were conducted in triplicate for each sample.

### Data analysis

Differentially methylated regions (DMRs) are found in biological samples collected under different conditions. In this study, DMRs were analyzed using the R package methylKit with default parameters of 1000 bp windows and 500 bp overlap, with a significance threshold set at *P* < 0.05 for differential screening.

Transcriptome data were extracted from the original images using Feature Extraction software (version 12.0.3.1, Agilent Technologies). The raw data were adjusted using quantile normalization and processed subsequently using Genespring software (version 14.8, Agilent Technologies). The normalized data were filtered to ensure that at least one probe set in each sample was identified as 100% accurate for subsequent analysis. Differentially expressed (DE) genes were identified with *P* < 0.05 as the screening threshold. These DE genes were subjected to Gene Ontology (GO) and KEGG enrichment analyses to identify the biological functions or pathways mainly affected by the DE genes. Finally, unsupervised hierarchical clustering was performed on the DE genes, and the expression patterns of these DE genes in different samples were visualized with the help of a heat map.

For protein quantification, data from at least two non-empty values across three repeated experiments in each sample group were selected for statistical analysis. Proteins exhibiting an expression difference greater than 1.5 fold (either upregulation or downregulation) and a *P* value < 0.05 were classified as differentially expressed proteins. For differential analysis, proteins with two or more non-null values in the data from one sample group and null values in another set of samples were included in the bioinformatics analysis. The results from both the significance and the presence/absence difference analyses were used to identify the differentially expressed proteins for further bioinformatics investigation.

## Results

### Results of methylation on chromosomes

**Fig. 1:**
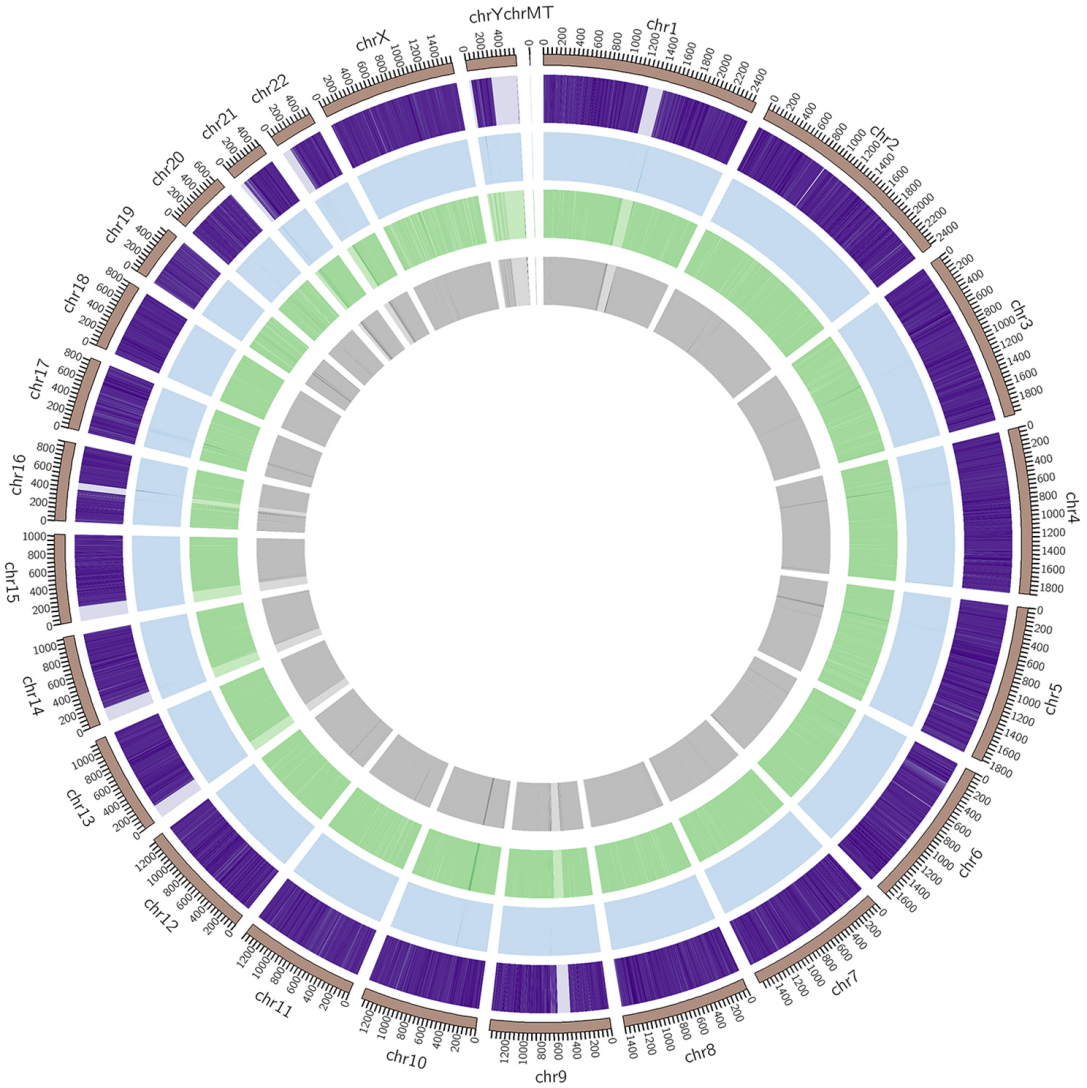
Results of methylation on the chromosomes of patients with pemphigus vulgaris. *Note*: (1) the outermost circle displays the chromosomal scale relative to its length; (2) the three middle circles (from outside to inside) represent the methylation levels of CpG (purple), CHG (blue), and CHH (green) in the corresponding chromosomal regions of the pemphigus vulgaris group; the darker the color, the higher the methylation levels; (3) the innermost circle represents the number of genes present in the corresponding region; the darker the color, the greater the gene density in this region

The results of the genome alignment analysis were used to determine the frequency of methylated CpG, CHG, and CHH sites across each chromosome. In mammals, methylation predominantly occurs on CpG sites, whereas in plants, methylation is more commonly observed at CHG and CHH sites [11]. To examine the specific distribution patterns of CpG, CHG, and CHH methylation on individual chromosomes, chromosome banding techniques were employed to visualize the data within defined chromosomal regions. The results of this analysis are presented in Fig. 1.

### Results of methylation in different gene segments

**Fig. 2:**
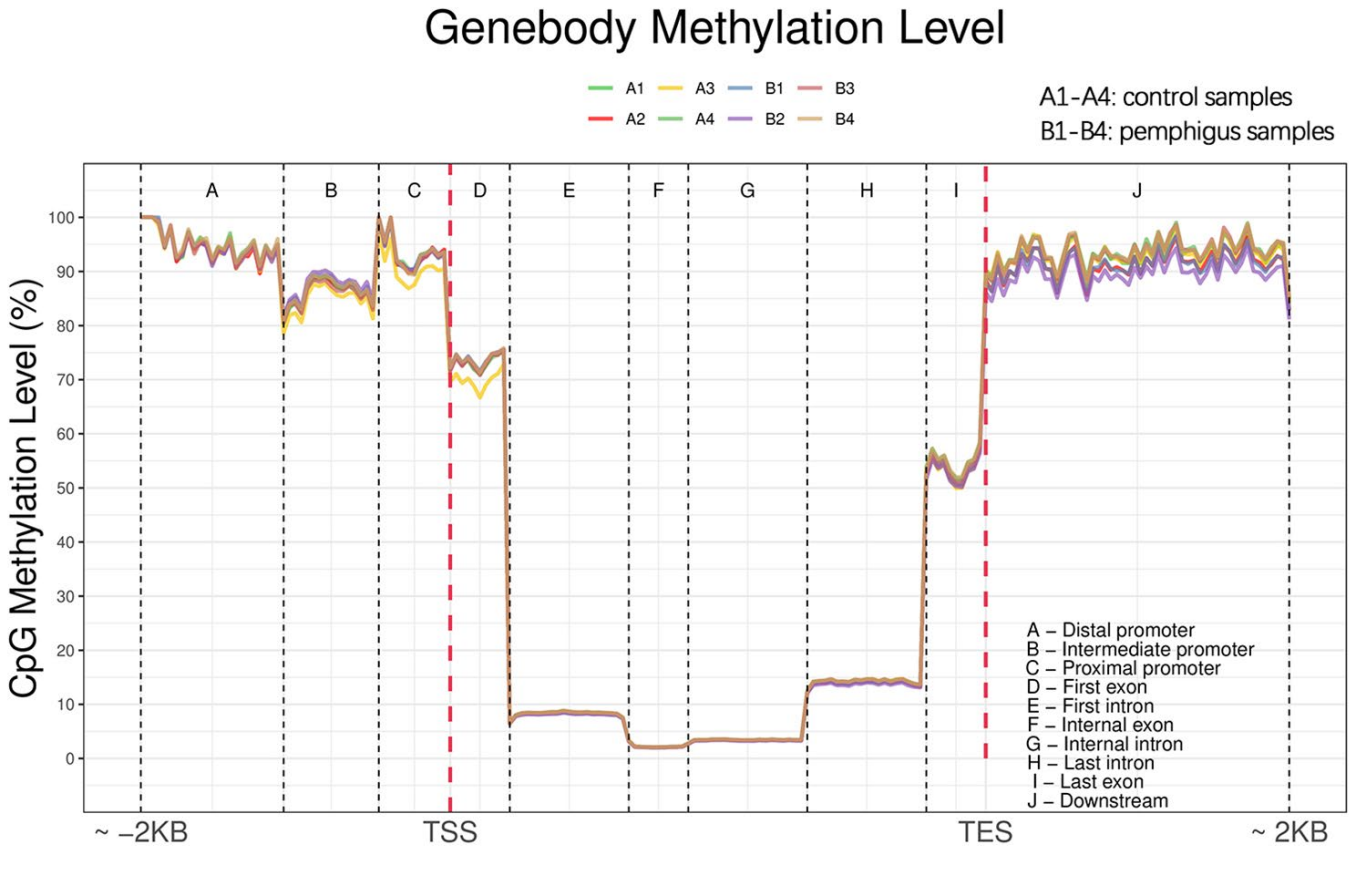
Results of methylation in different gene segments. *Note*: Compared to the control group, gene methylation is aberrant in the intermediate promoter, proximal promoter, first exon, and downstream regions in patients with pemphigus vulgaris. Methylation changes in the distal promoter, intron, intermediate exon, and final exon changes are relatively minor and mainly occur in CpG islands. Sample representations, control group: A1, A2, A3, A4; pemphigus vulgaris group: B1, B2, B3, B4

Changes in methylation across various functional elements of the genome were analyzed. The mean methylation levels of C sites were calculated within specific genomic regions, ranging from 2 kb upstream of the transcriptional start site (TSS) to 2 kb downstream of the transcriptional end site (TES). These regions were categorized into the promoter, exon, intron, and downstream areas. Based on the distribution of genetic structure methylation, the overall distribution of methylation across these functional regions was evaluated. Compared with the control group, there were notable aberrations in the methylation patterns of genes in patients with pemphigus vulgaris, particularly in the intermediate promoter, proximal promoter, first exon, and downstream regions. In contrast, the methylation levels in the distal promoter, intron, intermediate exon, and final exon regions exhibited relatively minor changes, as shown in Fig. 2.

### Analysis of DMRs

**Fig. 3:**
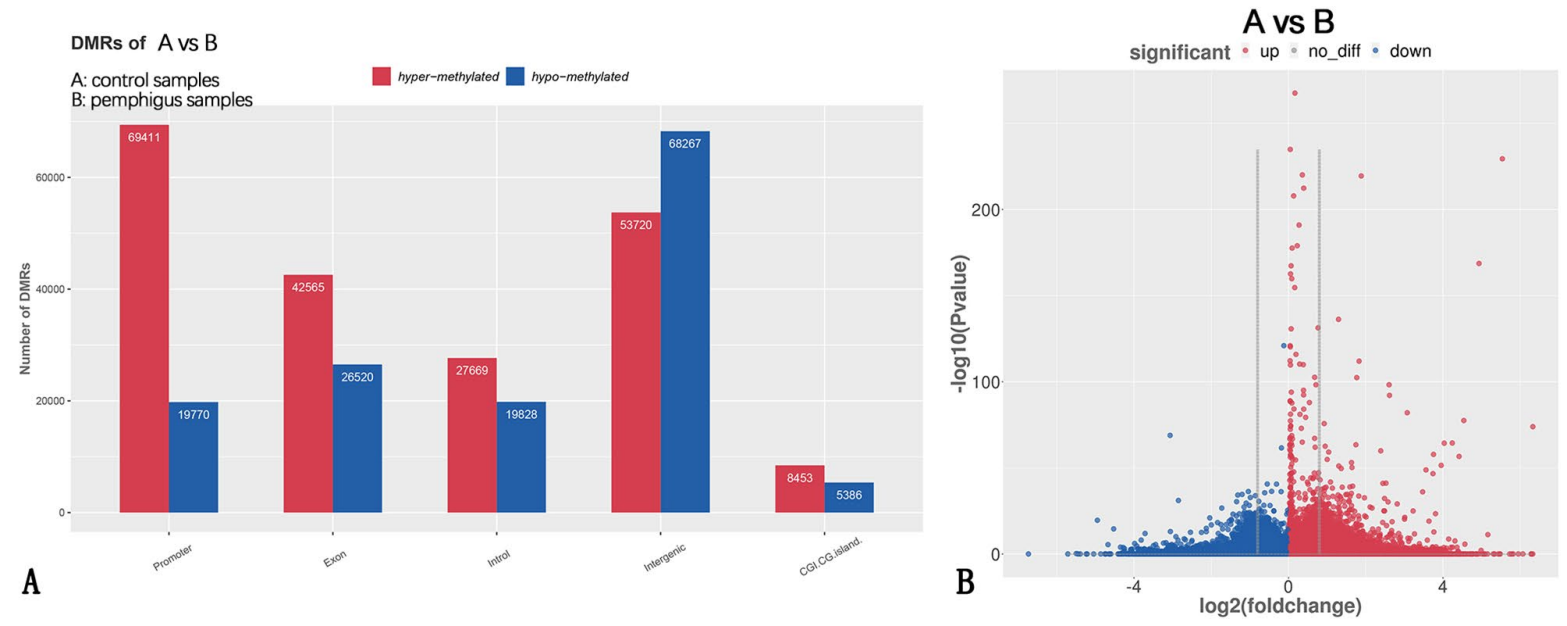
Analysis of DMRs. *Note*: (**A**) The *x*-axis indicates different gene functional regions, and the *y*-axis indicates the number of DMRs; red represents upregulated DMRs and blue represents downregulated DMRs. Compared to the pemphigus vulgaris group, hypermethylated regions in the promoter, exon, intron, and intergenic CGI regions were 69,411, 42,565, 27,669, 53,720, and 8,453, and the hypomethylated regions were 19,770, 26,520, 19,828, 68,267, and 5,386 in the control group. (**B**) The *x*-axis of the volcano plot of DMRs indicates the log of the methylation difference factor in DMR regions, and the *y*-axis indicates the log of the -log10 p value of difference analysis; red represents upregulated DMRs and blue represents downregulated DMRs. Significance was defined as a *P* value of < 0.05. Sample representations, control group: **A**, pemphigus vulgaris group: **B**

DMRs are key indicators of epigenetic changes and can be involved in the condition-specific regulation of gene expression, thereby affecting various biological processes [12]. The results showed that, compared with the pemphigus group, the hypermethylated regions in the promoter, exon, intron, and intergenic CpG islands were 69,411, 42,565, 27,669, 53,720, and 8,453, and the hypomethylated regions in these sane genomic segments totaled 19,770, 26,520, 19,828, 68,267, and 5,386 in the control group (Fig. 3, where group A represents the control group and group B represents the pemphigus group).

### Transcriptome data

**Fig. 4:**
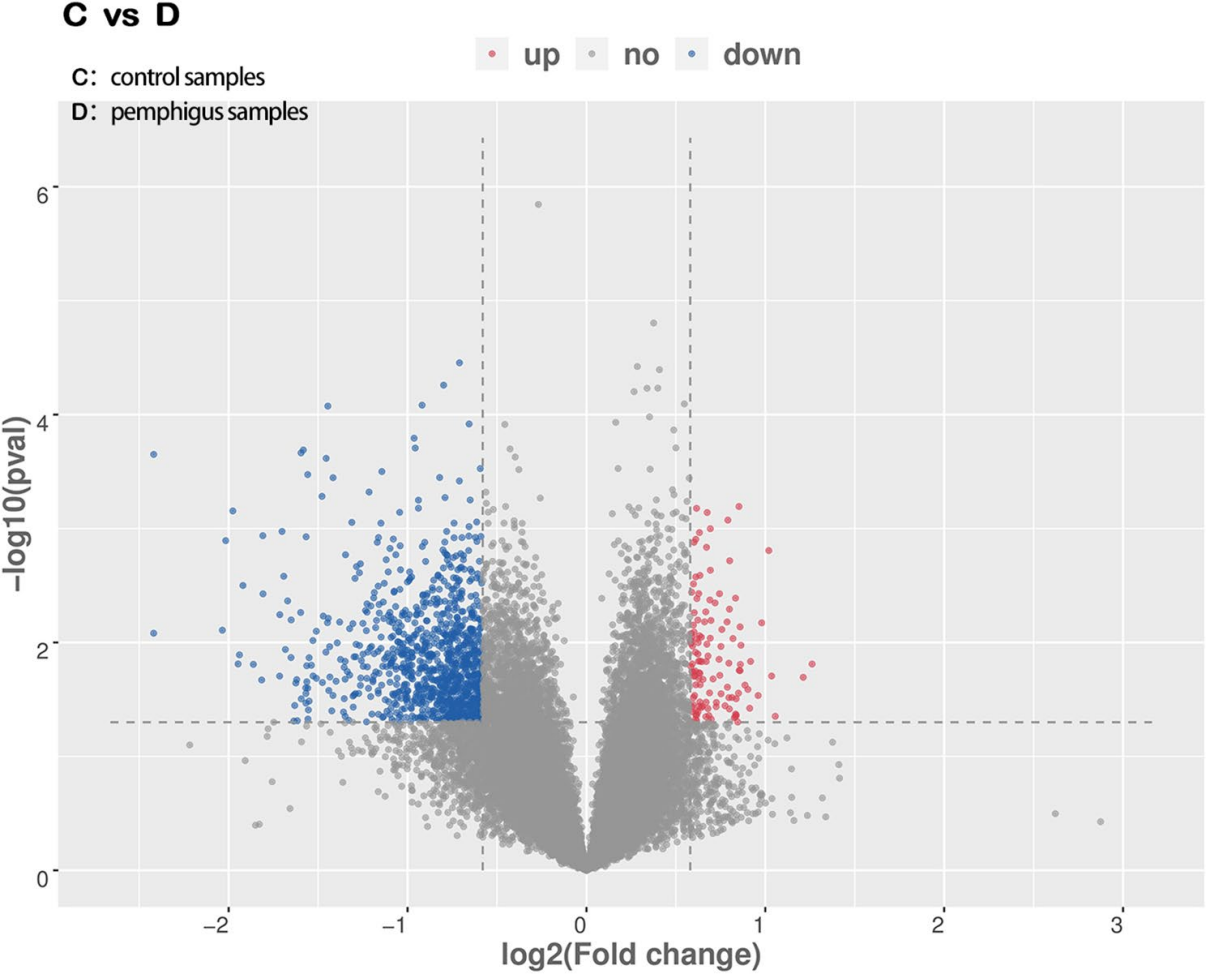
Volcano plot of transcriptome data. *Note*: Blue represents downregulated genes and red represents upregulated genes. Sample representations, control group: **C**, pemphigus vulgaris group: **D**

A total of 35,744 genes were identified in the transcriptome analysis, including 18,918 upregulated and 16,826 downregulated genes. Among these, 1,122 genes showed notable differences in expression: 998 genes were downregulated and 124 were upregulated (Fig. 4).

### Clustering analysis of differential mRNA expression levels

**Fig. 5:**
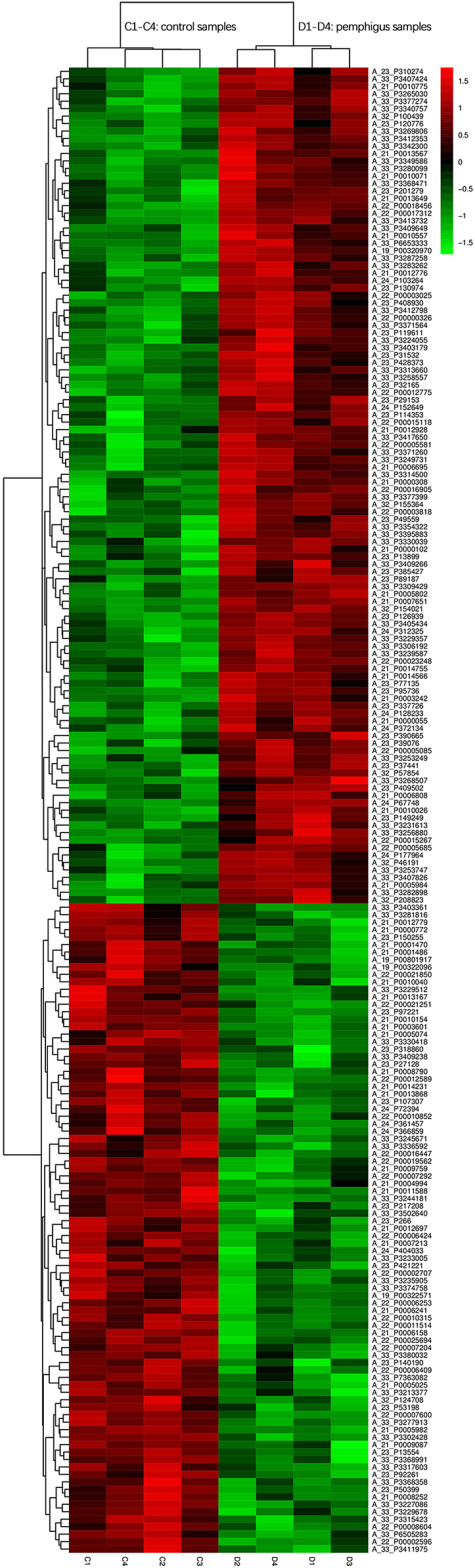
Clustering analysis diagram of DE mRNA expression levels. *Note*: The horizontal axis represents samples (control group: C1, C2, C3, C4, pemphigus vulgaris group: D1, D2, D3, D4). The vertical axis represents probe names. Color coding indicates transcription status as follows: green indicates transcriptional downregulation; red indicates transcriptional upregulation; transitions from green to black or red indicate increasing gene transcription levels

To illustrate the clustering patterns of differential gene expression, log10-transformed signal values were used, along with Z-score normalization to display signal intensities of differentially expressed (DE) genes. In the heatmap, the *x*-axis indicates samples and the *y*-axis indicates genes, with colors ranging from green to red indicating increasing levels of gene expression from low to high. Red corresponds to highly expressed genes, while green indicates genes with low expression levels. For this analysis, the top 200 DE mRNAs from the clustering analysis with the smallest *P* values were selected and used to constrict a hierarchical dendrogram (Fig. 5).

### Proteomics results

A total of 3,832 peptide fragments and 340 proteomes, including 219 DE proteins, were identified in the proteomics analysis. Quantitative analysis included data from proteins with at least two non-null values across three repeated experiments within each sample group. Proteins that met the screening criteria of a > 1.5-fold change in expression (upregulated and downregulated) and a *P* value < 0.05 were regarded as differentially expressed proteins. In the pemphigus group, 10 proteins were upregulated and 8 proteins were downregulated.

### mRNA analysis for methylation associations

**Fig. 6:**
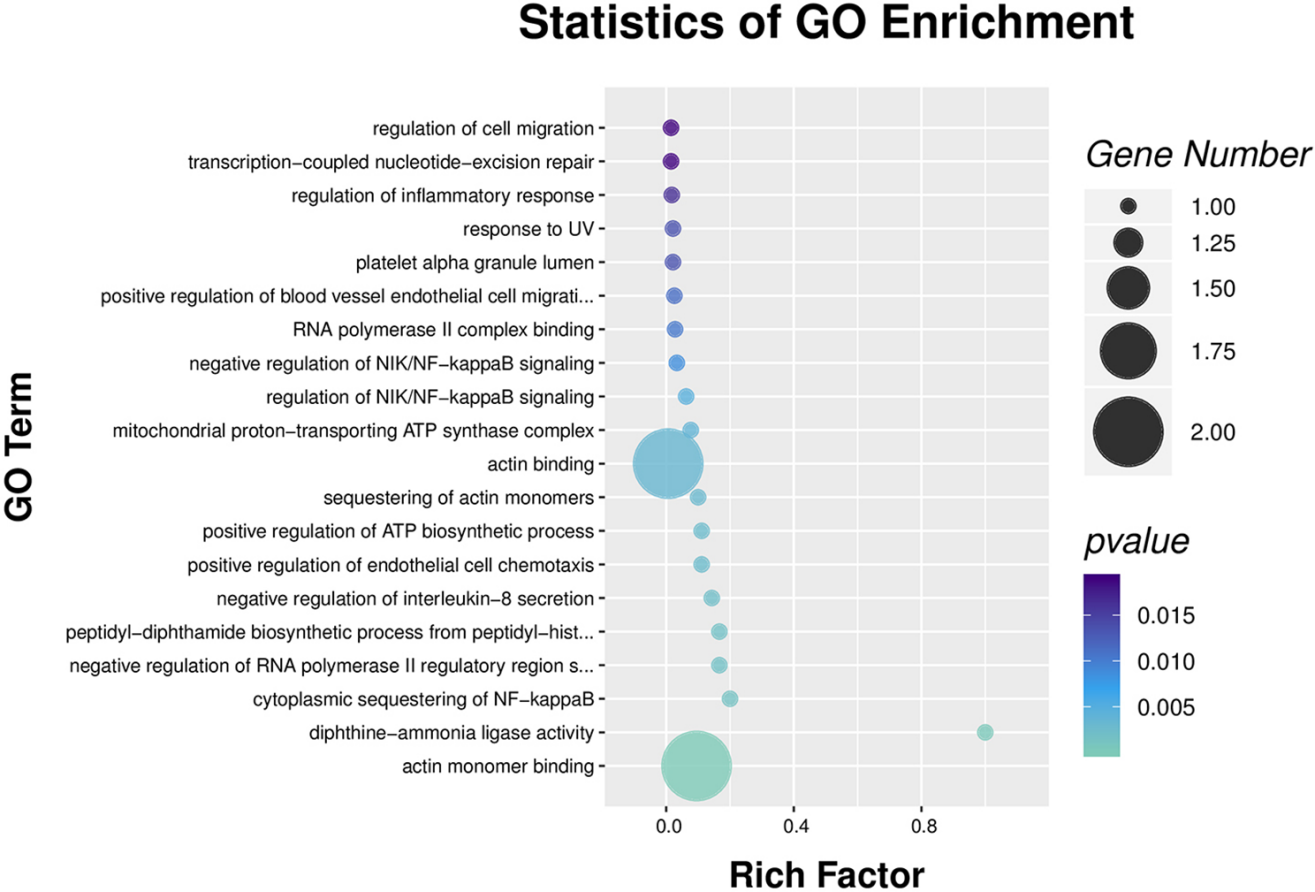
GO enrichment scatter diagram of mRNA analysis for methylation associations. *Note*: Methylation detection data were combined with mRNA and proteomics data for analysis. The results show that the differentially expressed genes are mainly involved in functions such as cell migration regulation, inflammatory response regulation, platelet activation regulation, NIK/NF-kappaB signaling regulation, actin binding, and other functions

The mRNA methylation analysis summary included data on all associations between methylation and gene expression, serving as a basis for subsequent data interpretation. GO annotation results showed that DE genes were mainly involved in biological processes such as regulation of cell migration, inflammatory responses, platelet activation, NIK/NF-kappaB signaling, and actin binding, among others, as shown in Fig. 6.

### mRNA and protein association analysis

To understand the relationship between mRNA and protein translation, databases such as Uniprot and Ensembl were used to build an association database. Due to regulatory variances, some mRNAs and proteins showed discordant expression patterns, suggesting potential negative correlations. A comprehensive association summary was generated to capture the significance and regulatory trends within each omics layer. Subsequent analyses were all based on this.

The summary of the omics association analysis highlighted notable relationships, detailing the regulatory direction and importance of each layer. Notably, the DE genes were mainly associated with biological processes such as platelet activation, innate immune response, inflammatory reaction, cytokine production, and the positive regulation of adhesion plaque assembly. In terms of cellular components, these genes were predominantly located in extracellular regions such as exosomes, the extracellular space, or the plasma membrane. Key molecular functions mainly included roles in protein binding, immunoglobulin binding, calcium ion binding, and receptor binding.

**Fig. 7:**
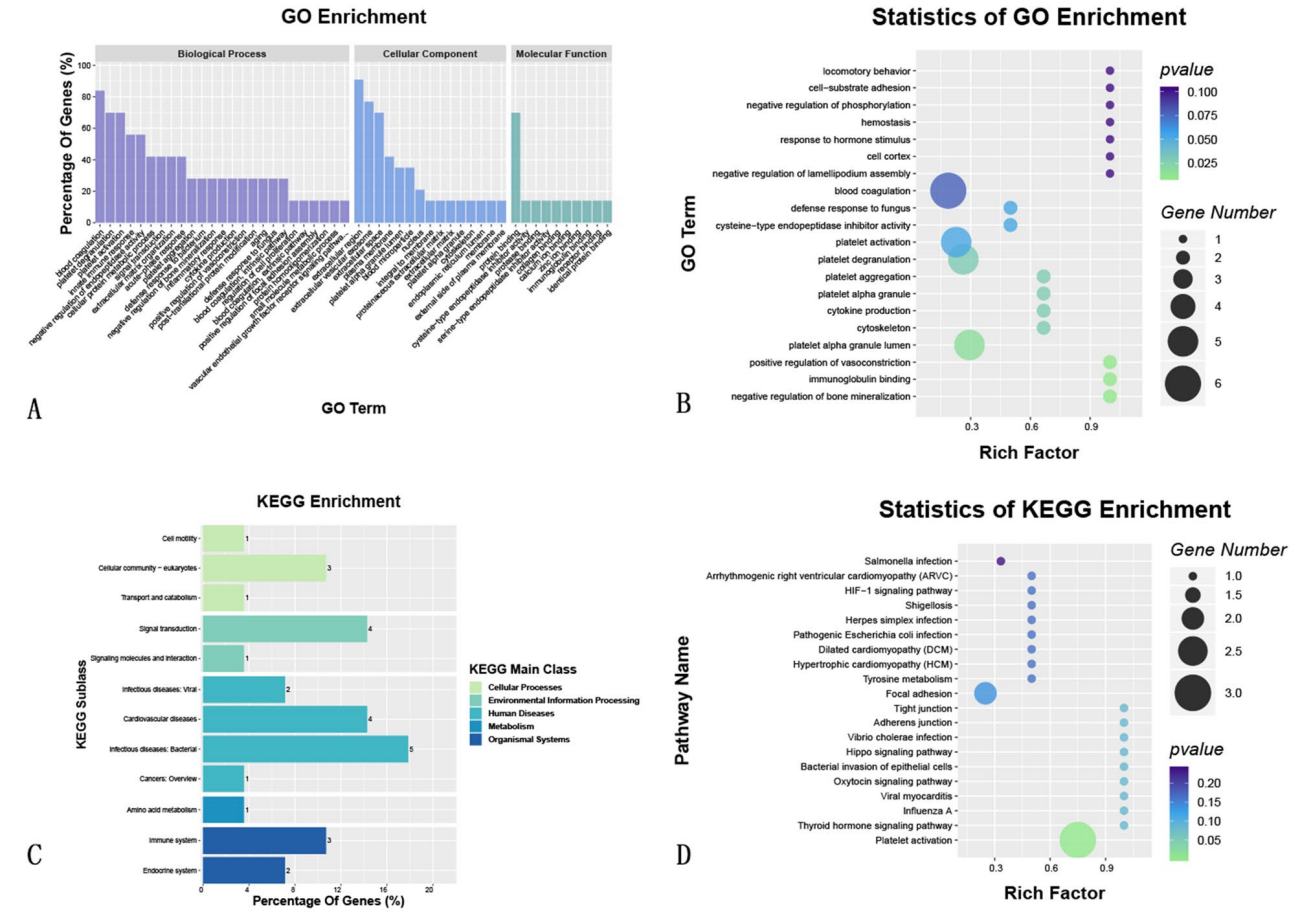
GO and KEGG enrichment and dendrogram of mRNA and protein association analyses

GO enrichment analysis showed that the DE genes between the two groups were mainly involved in biological processes related to platelet activation and coagulation, cellular adhesion structures, and immunoglobulin binding. Additionally, KEGG enrichment analysis indicated that these DE genes were primarily associated with pathways involved in bacterial infection, signal transduction, and immune system processes. Key pathways showing abnormal activity included the platelet activation pathway, adherens junction, tight junction, and infection pathways (Fig. 7).

## Discussion

Pemphigus vulgaris, as a chronic, autoimmune bullous skin disease, manifests with the formation of blisters that develop when autoantibodies compromise the desmosome adhesion structures among keratinocytes [13]. DNA methylation modifications play a vital role in the regulation of gene expression. In general, the higher the degree of CpG methylation, the lower the gene expression levels. In this study, analysis of peripheral blood DNA methylation from patients with pemphigus vulgaris revealed DMRs within the promoter, exon, intron, and downstream gene regions. Such DMRs may contribute to the differential expression of key genes involved in the disease, thereby influencing critical biological processes underlying its pathogenesis.

The GO enrichment analysis revealed that numerous aberrantly methylated genes were enriched in processes like protein, nucleotide, and nucleic acid binding, as well as the negative regulation of transcription by RNA polymerase II and calcium ion binding, among other molecular functions and biological processes. KEGG enrichment analysis indicated that these DE methylated genes were mainly enriched in metabolic pathways such as lipid, coenzyme and vitamin, amino acid, and carbohydrate metabolism, as well as in signaling pathways involving signal transduction, the immune system, and translation processes. Among these genes, 58 aberrantly methylated genes played a biological role via the mTOR signaling pathway.

The integrated analysis of methylation, mRNA, and proteomics data revealed involvement of DE genes in several key biological processes such as platelet activation, innate and inflammatory immune responses, cytokine production, and positive regulation of adhesion plaque assembly. Cellular components of interest were primarily extracellular, such as exosomes of extracellular vesicles, the extracellular space, and the plasma membrane. Molecular functions mainly included protein, immunoglobulin, calcium ion, and receptor binding. Central to the pathogenesis of pemphigus is the production of immunoglobulin (Ig) antibodies targeting keratinocyte surface proteins. The main target antigens are desmoglein 1 and desmoglein 3 of the desmoglein subfamily of cadherin adhesion molecules. IgG antibodies against desmogleins can destroy the adhesion function between keratinocytes, resulting in their separation from each other and blister formation. The analysis revealed disruptions in signaling pathways related to focal adhesion and tight junctions, suggesting that aberrant DNA methylation patterns in the peripheral blood of patients with pemphigus vulgaris may drive the abnormal expression of structural proteins crucial to intercellular junctions, thus compromising cell-cell adhesion.

GO enrichment statistical analysis revealed that DE genes from the two groups were mainly involved in biological functions such as platelet activation and coagulation, cellular structure adhesion, and immunoglobulin binding. KEGG enrichment analysis suggested that the DE genes were mainly concentrated in pathways related to bacterial infection, signal transduction, and immune system function. The KEGG enrichment analysis revealed that the main disrupted pathways were those involved in platelet activation, adhesion plaque, tight junctions, and infectious inflammation.

These results reveal the potential biological processes and pathways driving the observed differences in gene expression between the two groups. Such analyses enhance the current understanding of disease pathogenesis and also offer insights to guide the identification of potential drug targets and novel treatment strategies. For instance, if specific genes or pathways are found to be associated with specific diseases, these can become key targets for drug development. In addition, comparative analyses of gene expression patterns across various diseases or conditions can help identify potential therapeutic interventions.

In this study, an integrative analysis of methylation-associated transcriptomics and proteomics identified abnormally expressed genes, including *FGA* (fibrinogen alpha chain), *VWF* (von Willebrand factor), and *ACTG1* (*Homo sapiens* actin gamma 1), all of which are involved in platelet activation. In addition to their prominent function in hemostasis and thrombosis, platelets also serve as immune cells by initiating and regulating inflammation and immune responses [14, 15]. Platelets house a large number of immune-related molecules, and platelet activation can occur through stimulation by thrombin, chemokines, and microbial toxins [16, 17]. At the same time, adhesive and immune receptors—including P-selectin, CD 40 ligands, and toll-like receptors—are expressed on the surface of platelet cells, and they release soluble mediators such as chemokines, cytokines, and antimicrobial peptides [18, 19]. Platelets further interact with endothelial cells and leukocytes (including dendritic cells, T cells, B cells, neutrophils, monocytes, and natural killer cells) via both direct cell-to-cell contact and indirect mechanisms involving soluble mediator secretion [20]. 

Platelets play a prominent role in inflammatory skin diseases such as atopic dermatitis, contact dermatitis, and psoriasis through several pathological mechanisms. For instance, the formation of platelet-leukocyte complexes enhances leukocyte rolling on the endothelium, leading to the release of inflammatory mediators, including chemokines. This promotes the recruitment of leukocytes into inflamed skin, inhibits monocyte apoptosis, induces neutrophil cytophagy, promotes sensitization, and causes pruritus, collectively regulating inflammation [21–24]. Furthermore, platelets can also detect bacterial pathogens via interactions involving toll-like receptors, subsequently releasing antimicrobial peptides or clustering around bacteria to eliminate them [25]. Therefore, platelets are integral to both innate and acquired immune responses within the skin, engaging in complex interactions with leukocytes and endothelial cells.

In their retrospective study, Kridin et al. highlighted the crucial role of platelets in pemphigus vulgaris, noting that the mean platelet volume (MPV) of patients was reduced and that MPV was negatively correlated with severity of the disease. They also found that the MPV of patients with laryngeal involvement was lower than those without laryngeal involvement. Compared to healthy controls, MPV values were reduced in patients with pemphigus vulgaris [26]. In the present study, there was a notable increase in gene and protein expression levels related to platelet activation, suggesting that platelet activation plays an important role in the pathogenesis of pemphigus vulgaris.

The primary limitation of this study is the relatively small sample size, with only the minimum number of samples included to enable effective statistical analysis. Larger samples are necessary to validate these findings in future research.

In conclusion, numerous abnormally methylated genes were identified in this study, suggesting that changes in the transcription and expression of these genes may be associated with the pathogenesis of pemphigus vulgaris. Among these genes, *FGA*, *VWF*, and *ACTG1* were abnormally expressed, supporting the hypothesis that platelet activation may play an important role in the progression of pemphigus vulgaris.

## Data Availability

All data generated or analysed during this study are included in this article. Any use that does not involve commercial interests can be obtained directly by contacting the corresponding author.
